# Perioperative Myocardial Injury

**DOI:** 10.5152/TJAR.2023.22839

**Published:** 2023-02-01

**Authors:** Jack Brooker, Alparslan Turan

**Affiliations:** Department of Outcomes Research, Anaesthesiology Institute, Cleveland Clinic, Cleveland, United States

**Keywords:** Infarction, ischaemia, myocardial injury, perioperative, troponin

## Abstract

An increasing body of evidence suggests that a postoperative rise in cardiac troponin, even in the absence of other diagnostic criteria for myocardial infarction, is still associated with a range of postoperative complications including myocardial death and all-cause mortality. Myocardial injury after non-cardiac surgery is the term used to describe these cases. The true incidence of myocardial injury after non-cardiac surgery is unknown and likely underestimated. The strength of correlation with postoperative complications is also uncertain as are likely risk factors – though these are likely similar to those for infarction given the similar pathological mechanism. This review article seeks to summarise the literature which has been published over the preceding decades addressing these questions.

**Main Points**

- A postoperative rise in troponin even in the absence of signs or symptoms of myocardial ischemia is correlated with worse outcomes.
- The strength of correlation remains unclear due to the hetrogeneous surgical populations and troponin assays reported.
  Preventative measures include both primary and secondary prophylaxis such as ACE-inhibition, statins or antiplatelet treatments but the efficacy of these treatments remains unclear.
- Routine troponin monitoring after non-cardiac surgery in high-risk patients is indicated but there is unclear benefit in the general population. Management protocols are needed in patients with elevated troponin levels.

## Introduction

Myocardial injury after non-cardiac surgery (MINS) has become an increasing focus of interest in recent years. The lack of systematic assessment of cardiac markers and function in the postoperative period in patients undergoing non-cardiac surgery means the true incidence is unknown and likely much greater than initially estimated since many cases are asymptomatic. Recently, large multicentre trials have been conducted which suggest a troponin rise is indicative of myocardial injury occurring in approximately 20% of non-cardiac surgery cases, many of which are not accompanied by clinical symptoms or persistent electrocardiogram (ECG) changes.^1^ Furthermore, cases of clinically silent rises in postoperative troponins are associated with significant short- and long-term mortality.^2-4^ More than 300 million surgical procedures take place globally each year, meaning that the cumulative morbidity and mortality from MINS (both diagnosed and undiagnosed) is likely considerable.^5^

Myocardial injury after non-cardiac surgery refers to any injury and concomitant rise in biomarkers which frequently falls short of frank necrosis and infarction, as defined by the European Society of Cardiology, American College of Cardiology Foundation, American Heart Association, and World Heart Federation in their Universal Definition.^6,7^ There is evidence that many patients undergoing non-cardiac surgery sustain myocardial injury that fails to reach the threshold of infarction as defined by the Universal Definition, yet this injury remains a major influence on 30-day postoperative mortality. However, the quantification of MINS has previously been hampered by the inaccuracy, cost, and impracticality of routine perioperative testing.

As this has become more widely accepted in the field of perioperative medicine, attention has turned to the viability of screening and the utility of biomarkers to effectively quantify the number of patients affected by MINS as well as the strength of correlation between the results of these tests and the overall risk of perioperative morbidity and mortality. Per the statement from the American Heart Association, MINS constitutes elevated postoperative troponin that exceeds the 99th percentile of the upper assay reference limit which is presumed to be attributable to an ischaemic mechanism, with or without concomitant clinical signs or symptoms.^1^ Elevations in cardiac troponin (cTn) must be identified within the first 30 days after surgery but usually occur within the first 2 postoperative days.^1^ This is a significantly wider pool of patients compared to those encompassed by the Universal Definition (myocardial injury with a rise or fall of cTn above the 99th percentile of the upper reference limit and at least 1 of the following: ischaemic symptoms, new ischaemic electrocardiographic changes, development of new pathological Q waves on ECG, imaging evidence of myocardial ischaemia, or angiographic or autopsy evidence of coronary thrombus).^7^

## Challenges to Diagnosis

Intrinsic to the diagnosis of MINS is an elevated troponin (generally the 99th percentile upper assay limit per the 2021 statement from the American Heart Association).^6,7^ However, this must be in the absence of chronically elevated serum levels or probable non-cardiac causes numerous (e.g., renal impairment, severe pulmonary hypertension or infiltrative diseases such as amyloidosis), serial samples are indicated.^8^ Recommendations for those patients at risk of MINS, including patients over 65 or patients over 45 with documented coronary vascular disease, include a preoperative baseline cTn followed by a postoperative assay 48-72 hours after surgery. Where a preoperative cTn is not available, a repeat assay should be performed in the postoperative phase to ascertain an acute vs. chronic cause.^1^

A number of early prospective and some retrospective studies took place in the late 1990s and early 2000s in varying surgical populations. Studies varied in their surgical populations and endpoints. There were considerable disparities in the long- and short-term endpoints assessed, with some simply reporting all-cause mortality, whilst other authors reported cardiac-related death and others reported mortality as well as non-fatal myocardial infarction (MI) and MINS.^9^ Despite these significant differences in study design and endpoints, for both troponin and I and T, studies mostly reported a significantly elevated risk of death (odds ratio (OR) ranging from 4.7 to 14.9 for long-term mortality) and non-fatal cardiac complications such as MI, heart failure, and the need for coronary revascularisation.^9^

These early prospective and retrospective studies are limited by their small sample sizes, heterogeneous patient populations as well as the relative inaccuracy of the early troponin assays utilised and the inconsistent values defined as elevated. Nonetheless, they do collectively imply a relationship between mortality and elevated postoperative troponin in the absence of symptoms or sustained ECG changes suggestive of infarction.

Similar to earlier studies, a succession of prospective and retrospective studies emerged which sought to quantify the relationship between raised postoperative troponin and MINS. This later phase of studies marked the introduction of high-sensitivity troponin assays. Like the original phase of studies, the surgical populations varied (though orthopaedic patients predominate) as did the endpoints. The studies were largely united in reporting a significantly raised hazard ratio (HR) of death or major adverse postoperative events in patients with elevated postoperative troponin per study protocol (HR up to greater than 8.2) though some authors reported no correlation between perioperative troponin and mortality.^10,11^ Furthermore, preoperative elevations in troponins remained associated with an elevated OR for inpatient mortality.^11^ This was despite varying troponin assays and included those studies utilising newer high-sensitivity assays. These inconsistencies, together with the varying study designs and different time points used for troponin sampling in the postoperative phase and varying follow-up durations, make cross-study comparisons difficult.

The meta-analysis published by Ekeloef et al^4^ attempted to mitigate inter-study inconsistencies in methods and endpoints. Although these studies found that troponin was inconsistently associated with an increased likelihood of morbidity and mortality in their differing patient populations, Ekeloef et al^4^ found a 30-day mortality OR of 3.52 for those patients with elevated troponin, as well as a 2.53 OR for 1-year mortality. For secondary outcomes (major adverse cardiac events described earlier), the OR was 5.92 at 30 days and 3 at 1 year for those patients with elevated troponin measurements.

## Most Recent Studies

Ruetzler et al.^1^ in their 2021 Statement from the American Heart Association, recommend that all patients at high risk of MINS (e.g., those >65 or those >45 with a history of coronary or peripheral vascular disease) should have a baseline preoperative troponin level taken and then a postoperative level taken within 48-72 hours of surgery if the result would alter clinical management. Where a preoperative baseline measurement is unavailable, a second measurement should be taken following on from any elevated postoperative assay reading to establish if there is a rising or static pattern.^1^

A number of large studies in non-cardiac surgery patients were performed to establish the incidence of MINS and the association of raised postoperative troponin levels. Writing Committee for the VISION Study Investigators et al^2^ ran the VISION trial, which comprised 21 842 patients at multiple international centres. All participants underwent high-sensitivity troponin analysis 6-12 hours post-surgery and postoperative day (POD).^1-3^ They reported 17.9% of their overall cohort experienced MINS and 93% did not develop symptoms. However, elevated troponin in the postoperative phase was still correlated with an increased risk of 30-day mortality. Multivariate analysis showed a steady increase in HR as postoperative troponin levels rise and those with raised troponins but no ischaemic symptoms had a 3.2× HR of 30-day mortality.^2^ Puelacher et al^5^ included 2018 patients undergoing non-cardiac surgery in their international multicentre trial and required all participants to undergo both preoperative baseline troponin screening as well as postoperative screening. Using a rise of 14 ng L^−1^ as indicative of MINS, the authors reported an overall rate of 16%. However, only 18% of these patients displayed any symptoms of myocardial ischaemia. Multivariate analysis showed an overall HR of 2.7 for 30-day mortality in those patients with MINS and there was no significant difference in mortality between those MINS patients displaying criteria of infarction vs. those who did not (ischaemic symptoms, new ECG changes, or imaging evidence of loss of viable myocardium).

## Overall Incidence of Myocardial Injury After Non-Cardiac Surgery

Greater interest and the wider availability of large-cohort studies have increased the accuracy of MINS incidence estimates. A recent meta-analysis by Smilowitz et al^12^ included 169 individual studies with an overall cohort of 530 867 patients. Defining MINS as a rise and fall of cardiac biomarkers within 30 days of non-cardiac surgery that may occur with or without the clinical criteria necessary to fulfil the universal definition of myocardial infarction, they reported an overall incidence of 17.9%. This rose to 19.6% of patients when only the 139 studies utilising systemic screening of troponin (instead of clinically directed testing). Among this latter group of 30 studies, the incidence was only 9.9% which may imply a significant miss rate of cases when universal screening is not performed. Given the overall aetiology between MINS and MI does not differ—the distinction is simply a matter of degree-patient—dependent risk factors are predictable and borne out by published evidence. Male gender, increased age prior MI, renal disease, coronary artery disease, and heart failure were all associated with significant increases in the incidence of MINS after non-cardiac surgery.

## Aetiology of Myocardial Injury After Non-Cardiac Surgery

### Ischaemia

Ischaemia is critical to the development of MINS, and the absence of obvious non-ischaemic causes is necessary for the diagnosis.^1^ Since the distinction between MI and MINS is the absence of need for persistent ECG features or symptoms of myocardial ischaemia (frequently masked by sedation or analgesia), their underlying aetiology is very similar.

### Hypotension

Intraoperative hypotension has no common definition, but a mean arterial pressure (MAP) <65 mmHg is frequently used. Many patients experience a significant decline from their preoperative MAP, with 93% experiencing a 20% drop for a period of time.^13^ There is a known association between intraoperative hypotension and poor postoperative outcomes, including acute kidney injury, atrial fibrillation, delirium, and myocardial injury.^14-17^ Hypotension commonly occurs after surgery due to dehydration, blood loss, and the effects of general anaesthesia, with up to 48% of patients experiencing it after major non-cardiac surgery using MAP criteria of 60-75 mmHg.^18^ Periods of MAP <55 mmHg greater than 1 minute were associated with postoperative MI, with the OR correlated with the time spent below this MAP.^19^ Understanding the role of intraoperative MAP and its relationship with a number of postoperative outcomes including MINS is an ongoing area of research for the authors of this review article. The POISE 2 study demonstrated hypotension associated with clonidine use, which is known to be associated with cardiac injury.^20-22^

For example, Salmasi et al^20^ demonstrated that a MAP <65 mmHg or a MAP >20% below preoperative baseline was associated with progressively more severe myocardial injury with time spent below these thresholds intraoperatively. Importantly, intraoperative hypotension can occur at any time and may frequently go unnoticed by providers. It was reported by Sessler and Khanna^23^ that a third of all intraoperative hypotensions occur between induction and incision.

### Cardiac Failure

As a marker of atrial stretch, N-terminal hormonal brain naturetic peptide (NT-BNP) is a marker of a failing myocardium and long known as an objective marker of myocardial function and diagnosis of heart failure.^24^ Even in the absence of surgery or any externally driven stress response, advancing heart failure is associated with worsening myocardial perfusion to such a degree that troponin has been postulated as a useful biomarker for monitoring disease progression.^25^ The BNP has repeatedly been demonstrated as a reliable predictor of MINS, with Duceppe et al^26^ publishing a prospective study of >10 000 subjects which found that NT-proBNP of 100 to <200 ng L^−1^ was correlated with a 12% MINS incidence (adjusted HR, 2.29 [95% CI, 1.91-2.73]); 200 to <1500 ng L^−1^, with a 20% incidence (adjusted HR, 3.63 [95% CI, 3.12-4.21]); and ≥1500 ng L^−1^, with a 36% incidence (adjusted HR, 5.70 [95% CI, 4.69-6.92]).^1^ Thus, it is probably a better marker of cardiac fragility than a stress test.

### Pain and Surgical Stress Response

Surgery necessarily involves mechanical damage to tissue and accompanying physiological stress response, with increased catecholamine circulating in the bloodstream leading to sympathetic activation.^1^ In the setting of fixed perfusion limitations such as coronary vascular disease, this will lead to an increased supply–demand mismatch in myocardial perfusion, which, in turn, leads to ischaemia and MINS.^1^ In the immediate postoperative phase, pain is a major driver of adrenergic stimulation, leading to tachycardia, hypertension, and increased cardiac contractility, all of which places a significant metabolic demand on the myocardium. The link between postoperative pain and MINS was demonstrated by Turan et al^27^ in a large multicentre retrospective cohort analysis comprising 2892 patients undergoing non-cardiac surgery. Of these, 4.5% had elevated postoperative TnT (>0.003 ng mL^−1^) in the first 72 hours and there was a significant correlation with pain scores, with an HR of 1.12 for MINS with each unit increase in average pain.

### Anaesthetic Approach

The role that anaesthetic agents play in MINS is unclear. The ENIGMA-2 study did demonstrate that the addition of N_2_O to other anaesthetics did not increase the risk of myocardial injury, contrary to concerns.^28^ Volatile and total intravenous approaches appear to be similarly safe in non-cardiac surgeries with no difference in mortality or postoperative complications reported by Uhlig et al^29^ in their meta-analysis of 68 randomized, controlled trial (RCT). In cardiac surgery, however, the use of volatile anaesthetics appears to be associated with lower mortality and a composite of pulmonary and non-pulmonary complications, the latter including overall cardiac events and MI.

The mechanisms by which volatile anaesthetics appear to be cardioprotective, at least in cardiac surgeries, appear to be numerous but include a reduction in arterial and coronary perfusion pressure, a reduction in contractility and coronary vasodilation, thus minimising ischaemic damage.^30^ Despite this, it appears that anaesthetic depth does not have an impact on myocardial injury, as reported by the BALANCE study which found that anaesthesia to a BIS target level of 35 or 50 did not have any difference in either myocardial infarction or all-cause mortality at 1 year.^31^

Other anaesthetic approaches may be considered in the context of MINS. Presently, recommendations from the ICAROS study group are that neuraxial anaesthesia, if not contraindicated, should be used alone in hip/knee arthroplasty due to lower perioperative mortality compared to general anaesthesia. However, the evidence pertaining to cardiac complications (including or excluding infarction) is low.^32^ The REGAIN trial was the first pragmatic, randomised superiority trial comparing neuraxial and general anaesthetic approaches and their effect on postoperative mobilisation, finding no difference in overall mortality (a secondary outcome).^33^

### Surgical Approach

The effects of the surgical approach on MINS are unclear. Emergent surgery was shown in large cohort studies to have a greater correlation though this may be reflective of an overall sicker patient selection with greater intrinsic cardiac risk factors.^6^ Serrano et al^34^ identified vascular surgery on both univariate and multivariate analysis to be an independent risk factor for MINS though, again, it is unclear if this is reflective of a vasculopathic patient population with poor cardiovascular health. Meershoek et al^35^ did report that intraabdominal general surgery was also associated with a higher incidence of MINS. Other surgical factors such as laparoscopic versus open remain unclear with little or no primary research published which discusses the relative associations of these different approaches with MINS.

### Anaemia

Reduced oxygen carrying capacity is another mechanism for myocardial oxygen supply–demand mismatch and, due to perioperative blood loss and haemodilution, up to 40% of patients are anaemic after non-cardiac surgery.^36^ Turan et al^37^ identified a clear association between perioperative anaemia and MINS in 2 large-cohort retrospective studies. The first, a post-hoc analysis of 4 major trials including POISE-2, comprised 4480 patients undergoing major non-cardiac surgery who had routine baseline and postoperative TnT assessments in the first 7237. No patients whose lowest postoperative haemoglobin exceeded 13 g dL^−1^ experienced MINS, whilst 52/611 (8.5%) of patients whose minimum postoperative haemoglobin was <8 g dL^−1^ did, with an HR for MINS of 1.29 (1.16-1.42) for every 1 g dL^−1^ decrease in postoperative haemoglobin in a time-varying covariate analysis with those patients. The association between MI (third Universal Definition requiring elevated cTn plus one of persistent ECG changes and/or symptoms in the postoperative phase and/or evidence of coronary artery thrombus on angiography or autopsy^7^) and perioperative anaemia was demonstrated by a 7227 patient cohort in a post-hoc analysis of the POISE-2 trial.^38^ Among this cohort, 7.8% developed MI, with a composite outcome of non-fatal MI and all-cause mortality showing an HR of 1.46 for every 1 g dL^−1^ decrease in minimum postoperative Hb.^38^ The role of anaemia in perioperative MI injury was further characterised in the POISE-3 trial, which trialled the use of tranexamic acid (TXA) and statin therapy in patients undergoing major non-cardiac surgery. The trial found that the use of perioperative TXA significantly reduced major organ bleeding whilst being non-inferior on a composite of cardiovascular outcomes.^39^

## Prevention of Myocardial Injury After Non-Cardiac Surgery

### ACEi/ARB

The renin–angiotensin–aldosterone system is important in the pathological remodelling of cardiac vasculature so its inhibition in the perioperative phase may be cardioprotective provided it is titrated carefully and there are no periods of haemodynamic instability brought about by angiotensin converting enzyme inhibitor (ACEi) or angiotensin II receptor blocker (ARB) administration.

### Statins

A sub-study within the VISION trial focused on the perioperative utilisation of statins in non-cardiac surgery. A total of 2845 patients were treated with statins while 4492 patients acted as controls. The statin group had a significantly lower 30-day all-cause mortality, cardiovascular mortality, and MINS whilst there was no significant difference in MI or stroke.^40^ Conversely, a later study by the same lead author (LOAD trial) enrolled 648 statin naïve patients and randomised them either to a statin regimen comprising high dose atorvastatin followed by a 40 mg maintenance dose started within 12 hours or surgery or to a control group. They found that the statin group had no significant improvement in all-cause mortality, myocardial injury, non-fatal myocardial infarction, or stroke compared to the placebo group.^41^ Interest in the role of statins continues due to their plaque-stabilising effects. The POISE-3 trial includes a statin therapy arm though results are, as of yet, unpublished. Given the cardiovascular burden experienced by patients in the perioperative phase, it is biologically plausible that statin therapy can provide cardioprotective benefits through the prevention of ischaemia and, presumably, MINS though this is not clear at present.

### Beta-Blockers

The POISE-1 trial randomly assigned 8351 patients undergoing major non-cardiac surgery to receive extended-release metoprolol or placebo, starting hours before surgery and continuing for 30 days postop. The primary outcome was a composite of cardiovascular death, non-fatal myocardial infarction, and non-fatal cardiac arrest. Whilst the intervention group did experience less MI (HR = 0.73), this was more than nullified by the increased risk of all-cause mortality (HR = 1.33) and stroke (HR = 2.17).^42^

### Anticoagulation

Perioperative use of anticoagulation has been studied in relation to MINS in the MANAGE trial published in 2018. A total of 1754 patients undergoing major non-cardiac surgery and within 35 days of recorded MINS were randomly assigned to receive dabigatran for 2 years or placebo. Devereaux et al^43^ devised a primary efficacy outcome; occurrence of a major vascular complication, a composite of vascular mortality and non-fatal myocardial infarction, non-hemorrhagic stroke, peripheral arterial thrombosis, amputation, and symptomatic venous thromboembolism. They also devised a primary safety outcome, taking into account the possible negative side effects of long-term anticoagulation in these patients; a composite of life-threatening, major, and critical organ bleeding. The primary efficacy outcome was noted significantly more in the treatment group whilst there was no significant difference in HR for the safety outcome, implying a net benefit. The use of aspirin has been discussed in previous research with some authors reporting a reduced risk of MINS and others reporting either no significant difference or an increased risk of PMI.^44-47^ The POISE-2 trial enrolled over 10 000 patients undergoing major non-cardiac surgery and assigned them to receive either aspirin or placebo or clonidine and placebo. The primary outcome was a composite of mortality and non-fatal MI of which the study found no difference between aspirin or placebo groups, despite an increase in bleeding in the aspirin cohort.^48^ Smilowitz et al.^12^ in addition to reviewing the aforementioned studies, included 24 separate studies in their meta-analysis and found no significant change in the OR of MINS with the use of perioperative aspirin.

### Future Expectations

Further work is needed to establish the level to which MINS is affected by the MAP during surgery and which level is critical to the development of injury and poor outcomes. Traditionally, this was considered 65 mmHg; however, it is possible that blood pressure (BP) above this could be beneficial for the myocardium during surgery. Currently, Sessler et al (ClinicalTrials.gov Identifier: NCT04884802) are conducting the GUARDIAN trial, a multinational RCT assessing induction agents, pressers, and intraoperative BP control, with a “tight” control arm targeted at a MAP over 85 mmHg and a systolic over 110 mmHg, with a primary outcome, a composite of perfusion-related pathologies including myocardial injury. Furthermore, the POISE-3 trial comprises a hypotension avoidance arm, aiming for a MAP >80 mmHg, the outcome being a composite of vascular death, non-fatal MINS, non-fatal stroke, and non-fatal cardiac arrest in the first 30 days.

Whilst studies have reported little impact of anaesthetic agent or depth on the incidence of mortality or MINS (excepting cardiac surgery), questions remain about the impact of presser agents and induction agents, with the GUARDIAN trial hypothesising that propofol and norepinephrine provide better outcomes than etomidate and phenylephrine, respectively.

## Conclusion

Interest has grown steadily in postoperative myocardial injury in the last quarter of a century. The intrinsic physiologic stress, adrenal stimulation, and blood loss associated with major non-cardiac surgery mean that ischaemia and injury to the heart are predictable in a subset of the very large number of patients who undergo it every year. An increasing body of research with large patient cohorts suggests that approximately 15%-20% of non-cardiac surgery patients experience myocardial injury. More significantly, it appears that ischaemia associated with an increase in troponin levels, in absence of permanent ECG changes or symptoms of infarction, is associated with a significant increase in in-hospital and long-term mortality.

The last 25 years have seen a steady increase in the sensitivity of commercially available troponin assays, and with this, the universal screening of all non-cardiac surgery patients or at least specific cohorts of patients at increased risk of myocardial ischaemia has become possible. However, further research is required to describe the impact of available clinical interventions in the prevention of myocardial injury and the management of these patients in the postoperative period.
